# Don’t Anesthetize a Woman without a Witness

**Published:** 1873-07

**Authors:** H. C. Cressinger

**Affiliations:** Ashland, O.


### "DON'T ANESTHETIZE A WOMAN WITHOUT A WITNESS."

EDITOR REGISTER:—The article with the above caption in the editorial of the "Register" for June, called to mind very forcibly an incident in my own practice, wherein I narrowly escaped the loss of both reputation and practice. While studying for the practice of dentistry, my attention was called to an article in one of the current newspapers concerning a dentist in Pittsburgh, who had had charges preferred against him by a young lady, for criminal conduct while under the influence of chloroform. He was tried and convicted, and by *her testimony alone.* Whether he was guilty or not could not be known, as in such cases the testimony of a woman is final. I there resolved never to administer any anesthetic to a woman alone *under any circumstances whatever*, neither in twelve years practice have I done so; and with what wisdom the sequel will show.

In April of the year 1863, I administered chloroform to a middle-aged lady for the purpose of extracting teeth. The operation was performed in the presence of her own physician. In about a week subsequently, the physician told me that twice during the week had she asked him, in go od faith, whether I did not take improper liberties with her person; and so strong was the delusion produced by the chloroform during the operation. that nothing short of the testimony of an honest and faithful physician would have satisfied her.

Your note though short is timely; and so important do I

think it to be, that I am constrained to relate this fact in my practice, that younger practitioners need not stumble unwittingly. Much evil may be averted by strictly adhering to your advice: "Never to anesthetize a woman without a witness."

Yours very truly,

Ashland, O. H. C. Cressinger

